# MiR-125b promotes migration and invasion by targeting the vitamin D receptor in renal cell carcinoma

**DOI:** 10.7150/ijms.49328

**Published:** 2021-01-01

**Authors:** Xiyuan He, Shangfan Liao, Dongming Lu, Fabiao Zhang, Yingming Sun, Yongyang Wu

**Affiliations:** 1 Department of Urology, Affiliated Sanming First Hospital of Fujian Medicine University, Sanming, Fujian 365100, China.; 2 Department of Medical and Radiation Oncology, Affiliated Sanming First Hospital of Fujian Medicine University, Sanming, Fujian 365100, China.

**Keywords:** Renal Cell Carcinoma, Vitamin D Receptor, MicroRNA, MiR-125b

## Abstract

**Purpose:** To investigate the expression of miR-125b and vitamin D receptor (VDR) in renal cell carcinoma (RCC) and assess the biological function of miR-125b in RCC.

**Methods:** We used quantitative real-time polymerase chain reaction (RT-PCR) to detect the expression of nucleic acids and western blotting to analyze the protein abundance in RCC cell lines. MiR-125b mimic and inhibitor were employed to investigate the function and behavior of miR-125b in RCC cell lines. The relationship between miR-125 and VDR was verified using luciferase assays.

**Results:** Overexpression of miR-125b promoted migration and invasion and prevent cell apoptosis in ACHN cells. In contrast, miR-125b deficiency suppressed migration and invasion and induced cell apoptosis in 786-O cells. Luciferase assays indicated the interaction between miR-125b and VDR. In collected samples, miR-125b was significantly higher in RCC tissues and negatively correlated to VDR (r=-0.444, p=0.04).

**Conclusion:** MiR-125b displays an oncogene profile in RCC, patients with high expression of miR-125b should be a more frequent follow-up. MiR-125B may be a potential therapeutic target for RCC.

## Introduction

Renal cell carcinoma (RCC) accounts for approximately 3% of all human cancers and exerts the most lethal urological malignancies 1. Since the enormous progress in screening and treatment in the last decade, the prognosis is still poor. It is estimated that there were 73,820 new diagnoses and 14,770 deaths related to RCC in 2019 in the United States 2. Therefore, it is crucial to identify novel biomarkers and effective therapeutic targets in RCC.

Vitamin D receptor (VDR) is a nuclear class II receptor family that includes androgen receptor and estrogen receptor 3. 1,25-dihydroxy vitamin D3 is the specific ligand of VDR. Several studies revealed that vitamin D3 supplements would be a potential strategy to reduce the risk of RCC 4-6. On the other hand, VDR expression attenuates in RCC tissue, and loss-of-VDR predicted a poor prognosis in RCC 7-10. Of note, in our previous studies, we proved that VDR overexpression could inhibit RCC cell proliferation, migration, and invasion and promote apoptosis. Moreover, our findings demonstrated that VDR expression was associated with clinical-pathological characteristics in RCC 10-11. Altogether, we convinced VDR participated in oncogenesis in RCC.

MicroRNAs (miRNAs) are a class of small non-coding single-stranded RNAs with a length of 19-22 nucleotides and contribute greatly to human cancer by targeting mRNAs for cleavage or translational repression 12. In the present study, we searched TargetScan (http://www.targetscan.org/) and inferred VDR would be a target of miR-125b. Many studies have shown that mi-125b figures prominently regulate biological processes of human cancer via degradation or translational inhibition of specific target mRNAs 13-17. Many scholars reported the relevance of miR-125b expression and VDR in breast cancer 3,18,19. Although studies revealed miR-125b would be a potential oncogene* via* targeting VDR, it is still rare evident to display the direct binding of miR-125b and VDR.

Currently, the mechanism of miR-125b effects on the occurrence and development of RCC was still unclear. Herein, in this study, we aimed to investigate the function of miR-125b and VDR in RCC. Our results revealed that miR-125b was a potential oncogene *via* targeting VDR. MiR-125b deficiency inhibited RCC cell proliferation, migration, and invasion* in vitro*.

## Methods

### Cell culture

Human embryonic kidney 293T cells and 4 RCC cell lines, ACHN, A498, Caki-1, and 786-O, were purchased from the Type Culture Collection of the Chinese Academy of Sciences (Shanghai, China). Cells were cultured in Dulbecco's modified Eagle's medium (DMEM) (Gibco, Grand Island, NY, USA) supplemented with 10% fetal bovine serum (FBS) (Gibco, Grand Island, NY, USA), 100 µl/ml penicillin, and 100 mg/ml streptomycin. All the cells were incubated at 37 °C in a 5% CO_2_ atmosphere.

### Clinical sample collection

A total of 20 pairs of ccRCC and adjacent normal tissues were collected from Affiliated Sanming First Hospital of Fujian Medical University (Sanming, China) between May 2016 and May 2018. The Institutional Ethics Committee approved the study of Affiliated Sanming First Hospital of Fujian Medical University. Informed consent was obtained from all patients. The tumor samples were histopathologically diagnosed as RCC and stored in liquid nitrogen immediately after surgical resection until further use.

### Cell Transfection

ACHN or 786-O cells were seeded into 6-well plates at a density of 60%. Synthesized miR-125b mimic or inhibitor was transiently transfected into cells with Lipofectamine 2000 (Invitrogen, Carlsbad, CA, USA). MiR-125b inhibitors (sequence: 5'-UCACAAGUUAGGGUCUCAGGGA-3'), NC inhibitor (sequence: 5'-UCUACUCUUUCUAGGAGGUUGUGA-3'), miR-125b mimics (sequence: 5'-UCCCUGAGACCCUAACUUGUGA-3'), and NC mimic (sequence: 5'-UCACAACCUCCUAGAAAGAGUAGA-3') were purchased from GenePharma (Shanghai, China). The efficiency of miR-125b expression regulation was confirmed using qRT-PCR.

### RNA Isolation and qRT-PCR

Total RNA was extracted from the tissues and cells using TRIzol reagent (Invitrogen, Carlsbad, CA, USA) according to the manufacturer's protocol. For miR-125b detection, Taqman assays were employed, and U6 was used as an internal control. For mRNA analysis, total RNAs were reverse transcribed into cDNA using the RT MasterMix Kit (Abm). Then, qRT-PCR was performed using SYBR Green-I as the fluorogenic dye. The relative expression fold changes were calculated by the 2^-∆∆CT^ method.

### Cell proliferation assay

Cell proliferation was measured using the CCK-8 (Dojindo, Kumamoto, Japan) assay. Cells (786-O and ACHN) transfected with miR-125b mimic. The inhibitor was seeded into 96-well culture plates at a density of 1×10^4^ cells/well. The proliferation assay was carried out for 4 days, and cell growth was measured every 24 hours. Briefly, 10 μl of CCK-8 solution was added to each well, and the plate was incubated at 37 °C for 24 hours. Then, the absorbance of each sample was measured at 450 nm using a microplate reader. All experiments were performed in triplicate.

### Cell apoptosis assay

Cell apoptosis analysis was performed by flow cytometry using a fluorescein isothiocyanate (FITC) and propidium iodide (PI) kit (Vazyme, Nanjing, China). Cells were harvested after treatment with miR-125b mimic or inhibitor and washed with precooled PBS. The cells were mixed with 500 μL of binding buffer and 5 μL of FITC and PI and incubated for 10 minutes at room temperature (20 to 25 °C). Apoptosis rates were then measured by flow cytometry (FACS, Partec AG, Arlesheim, Switzerland). The percentage of apoptotic cells was calculated. Each experiment contained three replicates for each condition.

### Cell migration and invasion assays

Cell migration was measured using a Transwell chamber plate (Corning, Bedford, MA, USA). Briefly, 1×10^5^ cells were seeded onto the Transwell insert 48 hours after transient transfection. 20% of fetal bovine serum was used as a chemoattractant. After 48 hours of incubation at 37 °C, cells that did not migrate through the pores of the Transwell insert were manually removed with a cotton swab. The cells present at the bottom of the membrane were fixed in 4% polymethanol for 15 minutes and then visualized by incubation with 0.1% crystal violet for 20 minutes. Independent experiments were repeated three times. The invasion assay was performed similarly to the migration assay, with the only difference being that the upper layer of the Transwell chamber membrane uniformly covered the Matrigel. Two independent experiments were performed in triplicate for each experiment.

### miRNA target prediction and luciferase reporter assay

TargetScan was used to predict miRNAs that could potentially target VDR and identify possible binding regions. The VDR 3'-untranslated region (UTR) contained miR-125b binding sites. The dual-luciferase reporter system (Beyotime, Shanghai, China) was then used to verify luciferase activity. The VDR 3'-UTR cDNA sequence, including the mutant or wild-type miR-125b binding region, was amplified and cloned into the pGL3 luciferase vector (Promega, Madison, WI, USA). Next, 789-O cells were transfected with luciferase reporter plasmids and ether miR-125b inhibitor or NC using Lipofectamine 2000, according to the manuscript protocol. Then, the activity of luciferase was determined using a luminometer (Promega, Madison, WI, USA) and measured based on that of the empty pGL3 vector.

### Western blotting

VDR expression was assessed by Western blotting using standard protocols. Briefly, cells were harvested 48 hours after transfection, and lysates were prepared in radioimmunoprecipitation assay buffer. Equal amounts of protein, as determined by the Bradford Protein Assay (Beyotime, Shanghai, China), were separated by sodium dodecyl sulfate-polyacrylamide gel electrophoresis (SDS-PAGE) and transferred to polyvinylidene fluoride (PVDF) membranes (Beyotime, Shanghai, China). Five percent skim milk powder was added for 2 hours at room temperature. After incubation with a peroxidase-conjugated secondary antibody (Beyotime, Shanghai, China), the blot was developed using an enhanced chemiluminescence reagent and exposed to X-ray film to detect the labeled protein. β-actin served as a reference standard for protein expression.

### Statistical analysis

All statistical analyses were performed using GraphPad Prism 7 and SPSS 22, and all the data are presented as the mean ± SD. *P*<0.05 was considered to indicate a significant difference.

## Results

### miR-125b highly expresses in human RCC cell lines

**Figure 1:**
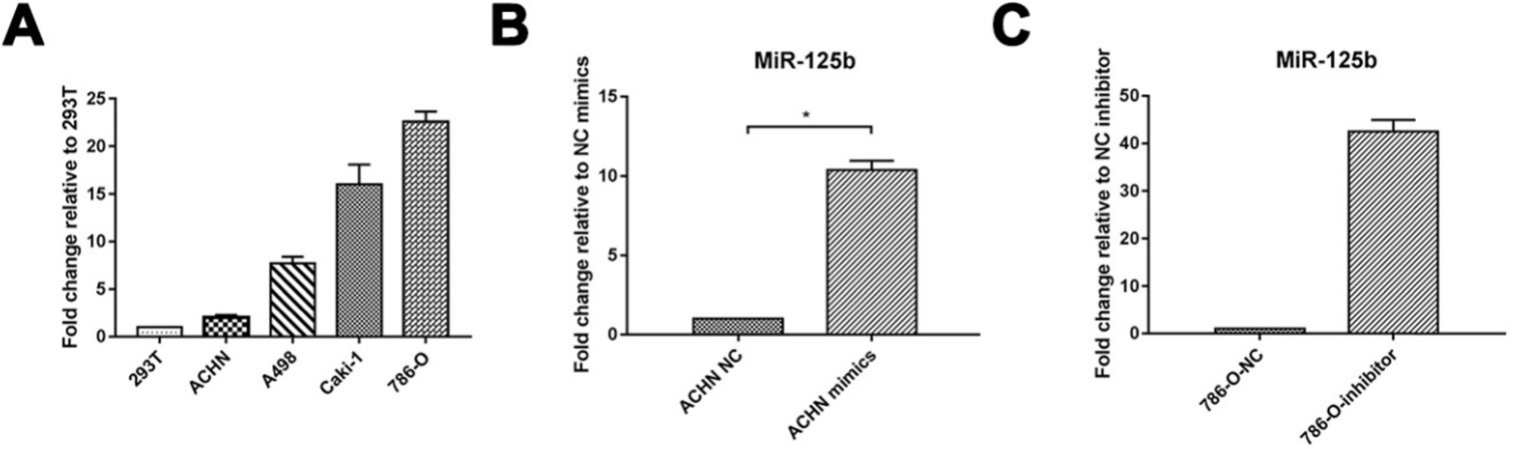
** miR-125b highly expresses in human RCC cell lines.** (**A**) MiR-125b expression level in human embryonic kidney 293T cell and RCC cell lines (ACHN, A498, Caki-1, 786-O). (**B**) The level of miR-125b in miR-125b mimic treated ACNH cells. (**C**) Expression of miR-125b in miR-125b inhibitor-treated 786-O cells. U6 was used as an internal control. *p<0.05, **p<0.01. NC=negative control.

We verified the expression of miRNA in RCC. 4 RCC cell lines, including ACHN, A498, Caki-1, 786-O, and embryonic kidney cell line 293T, were detected via Quantitative RT-PCR. miR-125b was increased in all the RCC cells compared to 293T. miRNA-125b expression in ACHN was deficient in RCC cell lines. We took it for mimics administration; meanwhile, 786-O displayed the highest expression of miR-125b and further selected for inhibitor administration (Figure 1A). As shown in Figure 1B and C, we successfully overexpressed miR-125b in ACHN cells and knockdown miR-125b in 786-O cells.

### miR-125b affects cell proliferation and apoptosis in RCC cells

We used the lipofectamine 2000 system to carry miR-125b mimics and inhibitors into ACHN and 786-O cells, respectively. Unfortunately, MTT results indicated both mimics and inhibitors were unable to affecting cell proliferation* in vitro.*

**Figure 2:**
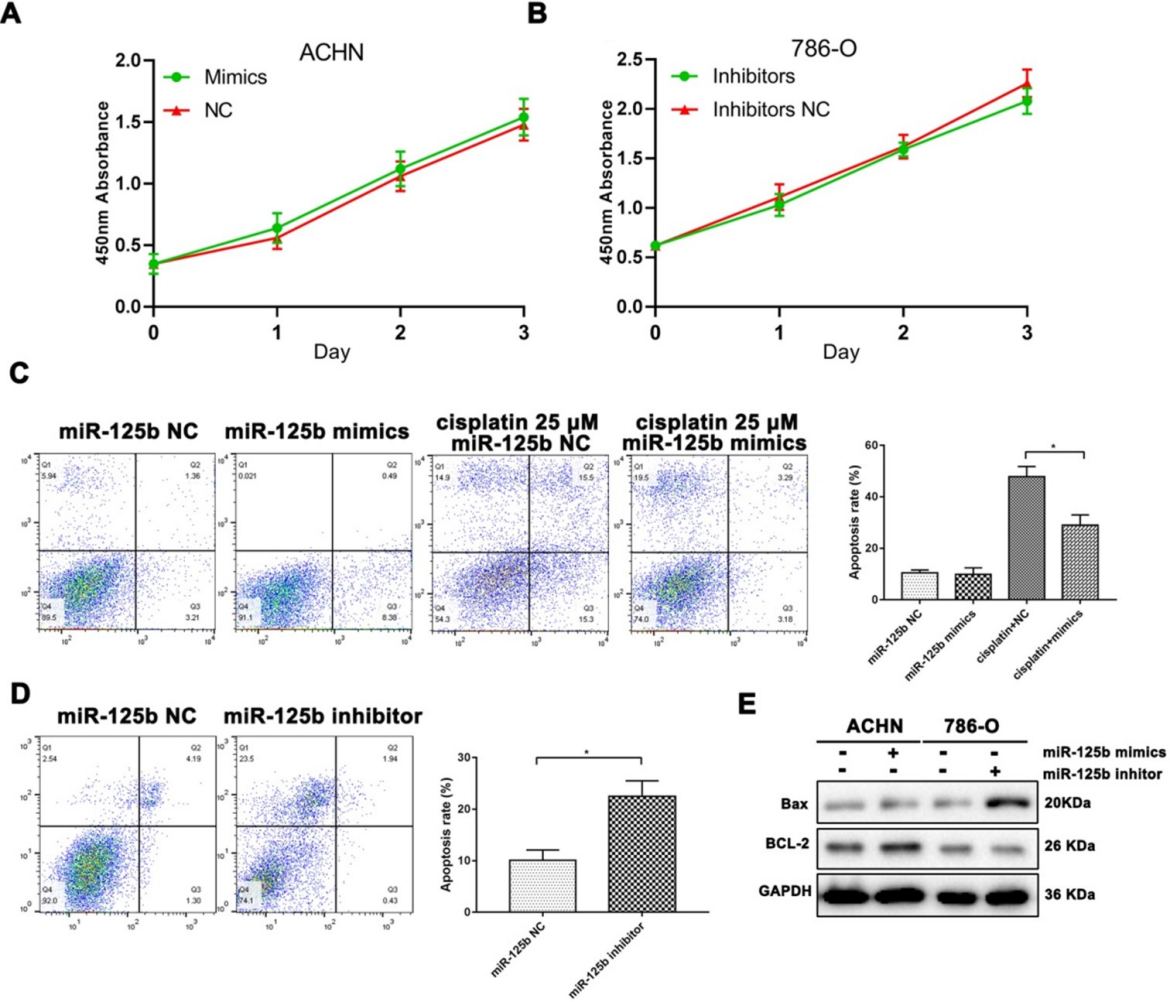
** miR-125b affects cell proliferation and apoptosis in RCC cells.** (**A**) The proliferation curve of ACHN cells with or without miR-125 mimics. (**B**) The proliferation curve of 786-0 cells with or without miR-125 inhibitor. (**C**) Annexin V-FITC and PI double staining illustrated the apoptosis rate of ACNH cells, grouping information as indicated. (**D**) Annexin V-FITC and PI double staining illustrated the apoptosis rate of 786-O cells, grouping information as indicated. (**E**) WB analysis of protein abundance of Bax and Bcl-2, GAPDH was as the loading control. *p<0.05.

However, we used cisplatin as a positive control of cell apoptosis. As shown in Figure 2C, miR-125b could prevent the cell from apoptosis under the pressure of cisplatin. Besides, as exhibited in Figure 2D, miR-125b inhibitors infection significantly increased the ratios of apoptosis in 789-O cells. Bax, a pro-apoptosis protein, was promoted after down-regulation of miR-125b; correspondingly, BCL-2, an anti-apoptosis protein, was enhanced in miR-125b overexpressed cells (Figure 2E).

### miR-125b facilitates migration and invasion of RCC cells

**Figure 3:**
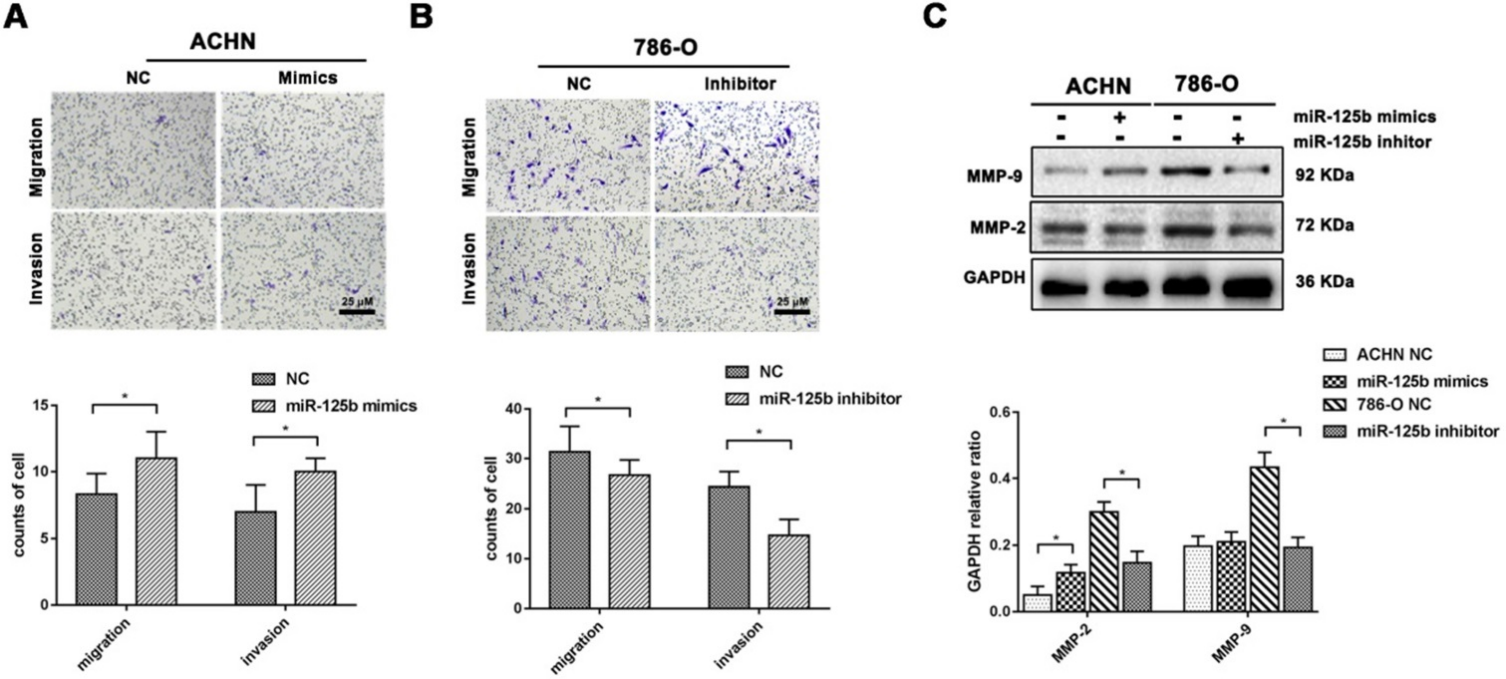
** miR-125b facilitates migration and invasion of RCC cells.** (**A**) Transwell assay for cell migration and invasion in ACHN cells. Representative images and quantitative information were illustrated. (**B**) Transwell assay for cell migration and invasion in 786-O cells. Representative images and quantitative information were illustrated. (**C**) WB analysis of protein abundance of MMP-9 and MMP-2, GAPDH was as the loading control. *p<0.05.

We performed a transwell assay evaluated the impact of miR-125b upon cell migration and invasion in RCC cells. We noted that mimics of miR-125b enhanced the cell migration and invasion ability in ACHN cells with up-regulating MMP-9; in contrast, cell migration and invasion were dramatically attenuated by miR-125b inhibitor with MMP-2 and MMP-9 attenuation in 789-O cells. These findings implied that miR-125b could facilitate cell migration and invasion in RCC cells (Figure 3A-C).

### MiR-125b targets and impairs the expression of VDR

**Figure 4:**
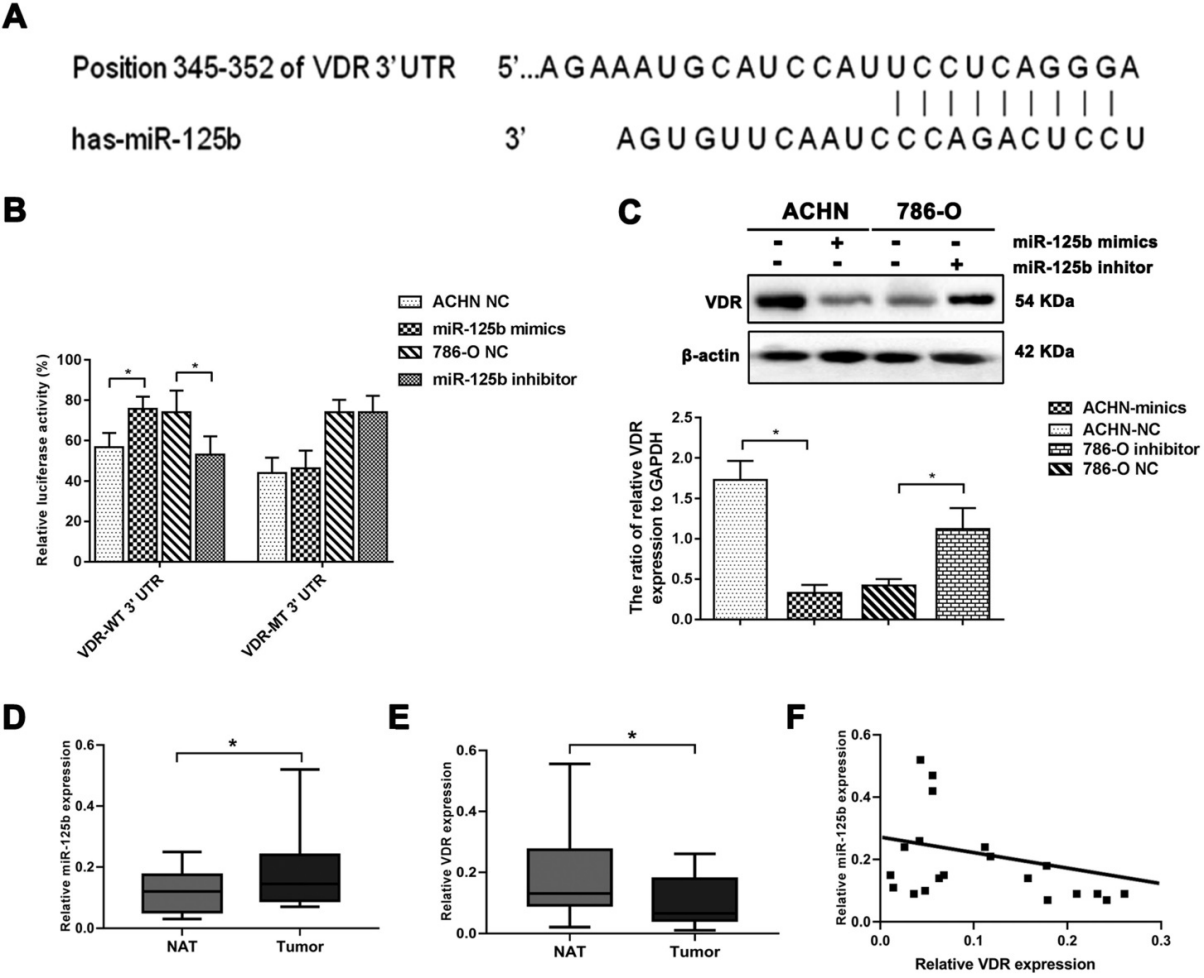
** MiR-125b targets and impairs the expression of VDR.** (**A**) The predicted binding region of miR-125b in the 3'-UTR of VDR. (**B**) Luciferase reporter assay indicated VDR might be a direct target of miR-125b. (**C**) WB analysis of protein abundance of VDR. β-actin was the loading control. (**D**) The expression of miR-125b increased in RCC tissues. (**E**) The expression of VDR decreased in RCC tissues. (**F**) Pearson correlation analysis of the association between miR-125b and VDR in RCC patients showed R= -0.44 (*p*=0.04). *p<0.05.

As previously described, we predicted VDR might be a potential target gene of miR-125b *via* TargetScan analysis (Figure 4A). We constructed a dual-luciferase reporter system to verify the direct binding of miR-125b and VDR. As displayed in Figure 4B, the miR-125b inhibitor significantly decreased luciferase activity in the cell with VDR 3'UTR miR-125b binding site wild-type plasmids. Besides, it was failed to impaired luciferase activity in the cell with mutation plasmids (Figure 4B). As display in Figure 4C, Western blotting indicated miR-125b negatively regulated the expression of VDR. These results indicate that VDR may be a direct target gene of miR-125b.

Moreover, we detected miR-125b and VDR expression in 20 RCC tissues and paired normal adjacent tissues. As shown in Figure 4D, E, RCC tissues expressed a higher level of miR-125b and lower level of VDR. Correlation analysis indicated miR-125b mRNA expression was negatively correlated with VDR expression (r=-0.444, *p*=0.04) (Figure 4F).

## Discussion

The expression of miR-125b has been reported to be altered in certain types of cancer. In normal tissue, miR-125b maintains in a low expression level 20, and increased during malignant hematopoiesis, suggesting its involvement in carcinogenesis 21. Still, Huang L et al. reported miR-125b was deficient in bladder cancer and impaired migration and invasion *in vitro* 22,23. Overexpressed miR-125b promotes migration, invasion, and metastasis in gastric cancer 24. High levels of miR-125 expression were associated with unfavorable clinical outcomes in non-small-cell lung cancer 25.

Similarly, miR-125b is sharply increased in chronic myeloid leukemia, and inhibition of miR-125b impairs proliferation with the G0/G1 phase arresting 26. These findings suggest that the role of miR-125b is essential in the tumorigenesis of certain tumors. Notwithstanding, the expression of miR-125b in different tumors varies largely.

Our finding presents similar results to the previous studies. Numerous studies have already proven the role of miR-125b in proliferation, apoptosis, and cellular differentiation in carcinogenesis 24-26. However, the mechanism by which miR-125b upregulation regulates migration and invasion in RCC remains elusive.

By bioinformatic analysis, we found the seed sequence of miR-125b was perfectly matched with the VDR mRNA 3'-UTR, and luciferase activity assay verified miR-125b could directly target the VDR 3'-UTR. Interestingly, 3'-UTR of VDR mRNA contains a miR-125b recognition element (MRE125b) containing 8 nucleotide elements, and that miR-125b could functionally recognize MRE125b. Furthermore, miR-125b mimics degrade the endogenous VDR protein level in breast cancer 3,18. In melanoma cell lines, the endogenous VDR mRNA level is inversely associated with the expression of miRNA-125b 27. In addition to these findings, our work reported that miR-125b should be a crucial regulator of VDR in RCC cells and promote the development and metastasis of RCC.

VDR, as a transcription factor, is one of the most studied tumor suppressor genes. Many studies reveal VDR participates in numerous physiologic processes including serum calcium regulation, cell proliferation and differentiation, and immunomodulatory functions 3. Our previous work demonstrated that VDR overexpression significantly suppresses RCC cell proliferation, migration, and invasion 11. Additionally, VDR could inactivate mutations, deletions, and promoter methylation to regulate cell proliferation and differentiation 4,28. In this article, we are failed to prove that miR-125b could regulate cell proliferation by targeting VDR. Multiple epigenetic interactions with VDR counteract cell proliferation, migration, and specific mechanisms require further study. Altogether, we concluded that miR-125b displays an oncogene profile in RCC, patients with high expression of miR-125b should be a more frequent follow-up. MiR-125b may be a potential therapeutic target for RCC.

## References

[1] Hsieh JJ, Purdue MP, Signoretti S (2017). Renal cell carcinoma. *Nat Rev Dis Primers*.

[2] Siegel RL, Miller KD, Jemal A (2019). Cancer statistics, 2019. *CA Cancer J Clin*.

[3] Singh T, Adams BD (2017). The regulatory role of miRNAs on VDR in breast cancer. *Transcription*.

[4] Bandera Merchan B, Morcillo S, Martin-Nunez G, Tinahones FJ, Macias-Gonzalez M (2017). The role of vitamin D and VDR in carcinogenesis: Through epidemiology and basic sciences. *J Steroid Biochem Mol Biol*.

[5] Muller DC, Scelo G, Zaridze D (2015). Circulating 25-hydroxyvitamin D3 and survival after diagnosis with kidney cancer. *Cancer Epidemiol Biomarkers Prev*.

[6] Muller DC, Fanidi A, Midttun O (2014). Circulating 25-hydroxyvitamin D3 in relation to renal cell carcinoma incidence and survival in the EPIC cohort. *Am J Epidemiol*.

[7] Obara W, Konda R, Akasaka S, Nakamura S, Sugawara A, Fujioka T (2007). Prognostic significance of vitamin D receptor and retinoid X receptor expression in renal cell carcinoma. *J Urol*.

[8] Blomberg Jensen M, Andersen CB, Nielsen JE (2010). expression of the vitamin D receptor, 25-hydroxylases, 1alpha-hydroxylase and 24-hydroxylase in the human kidney and renal clear cell cancer. *J Steroid Biochem Mol Biol*.

[9] Liu W, Tretiakova M, Kong J, Turkyilmaz M, Li YC, Krausz T (2006). Expression of vitamin D3 receptor in kidney tumors. *Hum Pathol*.

[10] Wu Y, Miyamoto T, Li K (2011). Decreased Expression of the epithelial Ca2+ channel TRPV5 and TRPV6 in human renal cell carcinoma associated with vitamin D receptor. *J Urol*.

[11] Chen Y, Liu X, Zhang F (2018). Vitamin D receptor suppresses proliferation and metastasis in renal cell carcinoma cell lines via regulating the Expression of the epithelial Ca^2+^ channel TRPV5. *PLoS One*.

[12] Farazi TA, Hoell JI, Morozov P, Tuschl T (2013). MicroRNAs in human cancer. *Adv Exp Med Biol*.

[13] Huang K, Dong S, Li W, Xie Z (2013). The Expression and regulation of microRNA-125b in cancers. *Acta Biochim Biophys Sin (Shanghai)*.

[14] Zhou JN, Zeng Q, Wang HY (2015). MicroRNA-125b attenuates epithelial-mesenchymal transitions and targets stem-like liver cancer cells through small mothers against decapentaplegic 2 and 4. *Hepatology*.

[15] Wang Y, Tang P, Chen Y, Chen J, Ma R, Sun L (2017). Overexpression of microRNA-125b inhibits human acute myeloid leukemia cells invasion, proliferation and promotes cells apoptosis by targeting NF-kappaB signaling pathway. *Biochem Biophys Res Commun*.

[16] Akhavantabasi S, Sapmaz A, Tuna S, Erson-Bensan AE (2012). miR-125b targets ARID3B in breast cancer cells. *Cell Struct Funct*.

[17] Zhao X, He W, Li J (2015). MiRNA-125b inhibits proliferation and migration by targeting SphK1 in bladder cancer. *Am J Transl Res*.

[18] Mohri T, Nakajima M, Takagi S, Komagata S, Yokoi T (2009). MicroRNA regulates human vitamin D receptor. *Int J Cancer*.

[19] Klopotowska D, Matuszyk J, Wietrzyk J (2019). Steroid hormone calcitriol and its analog tacalcitol inhibit miR-125b expression in a human breast cancer MCF-7 cell line. *Steroids*.

[20] He H, Wang L, Zhou W (2015). MicroRNA Expression Profiling in Clear Cell Renal Cell Carcinoma: Identification and Functional Validation of Key miRNAs. *PLoS One*.

[21] Shaham L, Binder V, Gefen N, Borkhardt A, Izraeli S (2012). MiR-125 in normal and malignant hematopoiesis. *Leukemia*.

[22] Huang L, Luo J, Cai Q (2011). MicroRNA-125b suppresses the development of bladder cancer by targeting E2F3. *Int J Cancer*.

[23] Wu D, Ding J, Wang L (2013). microRNA-125b inhibits cell migration and invasion by targeting matrix metallopeptidase 13 in bladder cancer. *Oncol Lett*.

[24] Chang S, He S, Qiu G (2016). MicroRNA-125b promotes invasion and metastasis of gastric cancer by targeting STARD13 and NEU1. *Tumour Biol*.

[25] Li Q, Han Y, Wang C (2015). MicroRNA-125b promotes tumor metastasis through targeting tumor protein 53-induced nuclear protein 1 in patients with non-small-cell lung cancer. *Cancer Cell Int*.

[26] Li Q, Wu Y, Zhang Y (2016). miR-125b regulates cell progression in chronic myeloid leukemia via targeting BAK1. *Am J Transl Res*.

[27] Essa S, Denzer N, Mahlknecht U (2010). VDR microRNA expression and epigenetic silencing of vitamin D signaling in melanoma cells. *J Steroid Biochem Mol Biol*.

[28] Marik R, Fackler M, Gabrielson E (2010). DNA methylation-related vitamin D receptor insensitivity in breast cancer. *Cancer Biol Ther*.

